# Case Report: Pharmacogenomics in clinical practice - a young male with medication-resistant depression and genetic variations in drug-metabolising enzymes

**DOI:** 10.3389/fpsyt.2025.1587875

**Published:** 2025-04-09

**Authors:** Cristina Beer, Annalese Semmler, Joanne Voisey

**Affiliations:** ^1^ Centre for Genomics and Personalised Health, School of Biomedical Sciences, Faculty of Health, Queensland University of Technology, Brisbane, QLD, Australia; ^2^ School of Clinical Sciences, Faculty of Health, Queensland University of Technology, Brisbane, QLD, Australia

**Keywords:** antidepressant response, medication-resistant depression, pharmacogenomics, CYP genes, PGx, depression, clinical practice, case report

## Abstract

Depression is a complex and heterogeneous mental health disorder affecting an estimated 280 million individuals worldwide. Although various antidepressant medications are available, a significant proportion of patients experience medication-resistant depression. This clinical case report highlights the critical importance of integrating pharmacogenomics into clinical practice, which is still not routinely done in many countries, through the detailed examination of a mid-20s male patient diagnosed with medication-resistant depression. Genetic analysis revealed specific variations in the cytochrome P450 genes, namely CYP2D6, CYP2C19, and CYP1A2, which are crucial for drug metabolism. By investigating the impact of these genetic variations on the patient’s treatment response, we provide evidence-based recommendations for adjusting antidepressant medications based on the individual’s unique pharmacogenomic profile. As demonstrated in the case report, this ultimately results in a positive clinical outcome and would have been advantageous to implement earlier as part of the patient’s management.

## 1 Introduction

Depression, a multifaceted and diverse mental health condition, impacts millions of individuals globally (1). Of the estimated 280 million individuals worldwide suffering from major depressive disorder (MDD) (1), only around half of these individuals will have an adequate response to an initial trial of antidepressant medication (2), the mainstay of current treatment for moderate to severe depression. The remainder of patients have a high chance of experiencing medication-resistant depression (MRD), where standard treatment approaches fail to achieve remission or adequate symptom relief after trialling at least two antidepressants at an appropriate dosage and duration (2). MRD poses a significant challenge for clinicians, necessitating a personalised approach to identify optimal treatment options for individualised patients. In recent years, pharmacogenomics (PGx) has emerged as a tool that can provide insights into how an individual’s genetic makeup influences their response to medications. By leveraging genetic testing, clinicians can tailor treatment regimens according to their patient’s specific genetic variations, potentially improving therapeutic outcomes in MRD cases. Clinicians are largely still unsure as to how to best integrate and utilise pharmacogenomics in clinical practice despite growing evidence for its clinical utility in the management of mental health conditions (3).

### 1.1 Understanding medication-resistant depression

The causes of MRD are multifactorial, including genetic, environmental, and psychological factors (2). Interindividual variability in drug response can also be related to age, gender, lifestyle factors, obesity, and ethnicity, all of which influence the process of drug metabolism. The management of depression is largely still based on the landmark Sequenced Treatment Alternatives to Relieve Depression (STAR*D) report (4), which broadly recommends either increasing the dose of an antidepressant medication or sequential trial of another class of antidepressant when treatment response has been suboptimal (and/or addition of adjuvant therapy - not discussed further here) until such point that treatment is effective and/or deemed unsuccessful (physical therapies are subsequently trialled such as electroconvulsive therapy or the newer transmagnetic stimulation).

In terms of the genetic causes of MRD, it has been found that genetic variations in specific genes encoding drug-metabolising enzymes, drug transporters, and drug targets can significantly influence the effectiveness and tolerability of antidepressant medications (5).

### 1.2 PGx and its application in depression treatment

PGx involves the study of how an individual’s genetic variations influence their response to medications. By identifying specific genetic markers, clinicians can predict an individual’s likelihood of responding positively or negatively to various medications, allowing for a more personalised and precise approach to treatment. In the context of depression, PGx testing provides valuable information about an individual’s genetic variations, in particular, in the cytochrome P450 genes which encode drug metabolising enzymes, as these greatly impact the efficacy and side effect profile of anti-depressant medications (6).

Several countries around the world now offer routine PGx testing in clinical practice, with the US and UK leading the way. The US Food and Drug Administration (FDA) was the first to offer approved PGx labelling of medications with other countries following suite. Large PGx databases and consortiums, for example, the Pharmacogenomic Knowledge Base (PharmKGB), Clinical Pharmacogenetics Implementation Consortium (CPIC), The Canadian Pharmacogenomics Network for Drug Safety (CPNDS), The French National Network of Pharmacogenetics (RNPGx) and the Dutch Pharmacogenetics Working Group (DPWG), currently offer clinical practice prescribing recommendations to guide medication dosing where genetic variations or polymorphisms exist (7–11). A polymorphism refers to the existence of genetic variations within a population group, which may include a variation in gene copy number or in single nucleotides within a specific position of a DNA sequence that encodes a protein e.g. enzyme, transporter, or receptor.

### 1.3 Genetic variations and antidepressant metabolism

Several cytochrome P450 (CYP) enzymes, including CYP2C19, CYP2D6, CYP1A2, and CYP2B6, play significant roles in the metabolism of antidepressant medications (6). Genetic variations in genes encoding these enzymes can result in altered drug metabolism, leading to variations in drug efficacy and side effect profiles. As an example, individuals who are poor metabolisers of CYP2D6 may experience increased plasma drug concentrations and may be at higher risk of adverse effects when treated with certain antidepressant medications which are metabolised primarily by this enzyme. On the other hand, ultrarapid metabolisers of CYP2D6 may require higher doses to achieve therapeutic concentrations as the medication is rapidly broken down in the body resulting in reduced plasma concentration potentially leading to reduced efficacy.

CYP genetic test results are commonly reported as the combination of the inherited maternal and paternal star (*) alleles, which is referred to as a diplotype (e.g., CYP2D6*1/*2). The predicted phenotype is influenced by the expected function of each reported allele in the diplotype, that is, in the case of the CYP450: poor metaboliser, intermediate metaboliser, extensive (or normal) metaboliser, or ultrarapid metaboliser. This phenotype may also be influenced by other factors including other drugs the patient may be taking (referred to as phenoconversion) (12). Understanding the full patient medication history is therefore important.

This case report focuses on a male in his mid-20s with MRD and explores the implications of his genetic variations in *CYP2D6*, *CYP2C19* and *CYP1A2* on clinical response to various anti-depressant medications trialled. Additionally, adjustments to his medication regimen based on PGx information will be discussed. Note that these single nucleotide polymorphisms (SNPs) were the only ones able to be tested in the clinically available panel at the time of testing. Other SNPs exist that have been associated with antidepressant medication clinical efficacy including *CYP2B6*, the *ABCB1* transporter gene, *HTR1A/2A* serotonin receptor genes, and the *SLC6A4* serotonin transporter gene.

## 2 Case description

**Figure 1:**
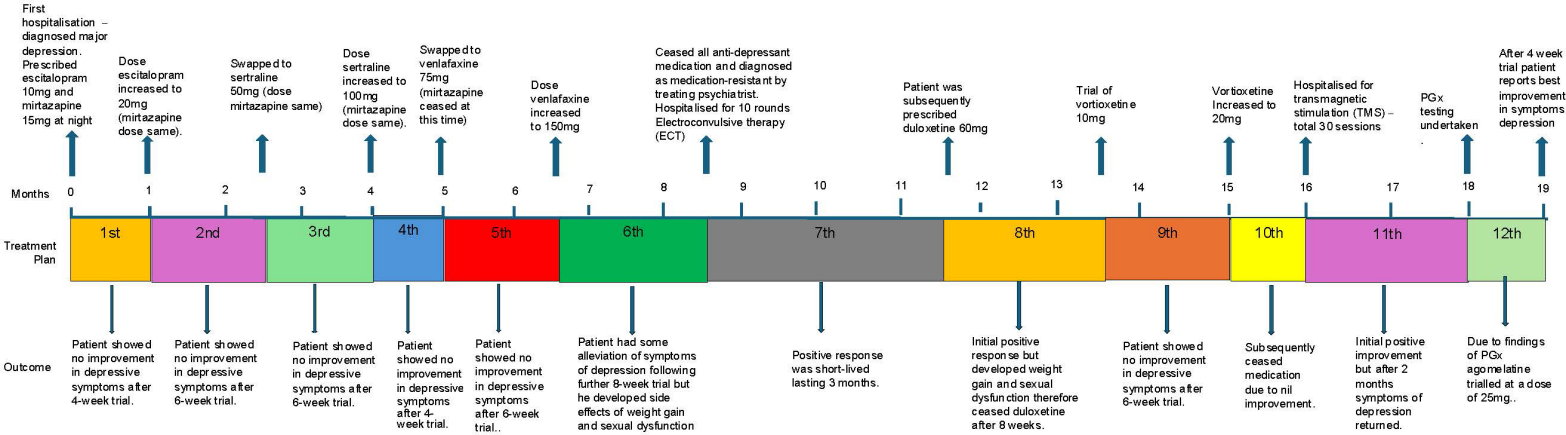
Timeline of patient treatment.

The patient, a man in his mid-20s, of European ancestry, who has given consent to publish his case, has been diagnosed with MRD. He first presented following a major depression episode in which he was hospitalised for a suicide attempt. Over the course of 18 months, he has trialled various antidepressant medications from different classes, all except the last medication trialled following PGx testing, have either provided, at most, only partial relief of symptoms and/or lead to adverse reactions, and early cessation. Along with medication he has also trialled electroconvulsive therapy (ECT) and transmagnetic stimulation (TMS), both of which are reported to have failed. He has been under the care of several psychiatrists and a general practitioner. Along with major depression, he has been diagnosed with other mental health co-morbidities, including post-traumatic stress disorder, generalised anxiety disorder, obsessive compulsive disorder, autism spectrum disorder, and chronic insomnia. He has no other medical co-morbidities, is not taking any other medications, is a non-cigarette smoker, does not drink alcohol, nor does he partake in recreational illicit drug use. There is a family history of mental health conditions with one sibling experiencing symptoms of major depression. During the routine clinical work-up, there were no organic, haematological or biochemical abnormalities detected. A treatment timeline for this patient is provided in **Figure 1** and outlined below. Note, along with the medications listed in **Table 1**, the patient has been actively engaged in psychotherapy with a registered psychologist. Clinical assessments were performed using DSM-5 criteria.

**Table 1:** Medications trialled by patient (in order prescribed), clinical response and recommended adjustments based on PGx profile.

Medication	Dosage#	Clinical Response	Main CYP450 enzyme substrate (patient phenotype in brackets)	Possible Effect of PGx on Plasma Concentration	Recommended dosage adjustments based on PGx****
Escitalopram	10mg, 20mg	Ineffective	CYP2C19 (ultrarapid)	Reduced	Increased dose recommended
*Mirtazapine	15mg	Ineffective, side effects	CYP2D6 (intermediate) CYP2C19 (ultrarapid)	Possibly increased Reduced	No current recommendation exists
Venlafaxine	75mg, 150mg	Partial, side effects	CYP2D6 (intermediate)	decreased primary metabolite (desvenlafaxine): parent drug (venlafaxine) ratio.	Insufficient evidence for intermediate metabolisers***
Sertraline	50mg, 100mg	Ineffective	CYP2C19** (ultrarapid)	Reduced	Initiate therapy with recommended starting dose. An increased dose may be required to achieve therapeutic benefit
*Duloxetine	30mg, 60mg	Partial, side effects	CYP2D6 CYP1A2	Possibly increased Usual	No current recommendation exists
Vortioxetine	10mg, 20mg	Ineffective, side effects	CYP2D6	Increased	Initiate therapy with recommended starting dose.
Agomelatine	25mg	Effective, nil side effects	CYP1A2	Usual	No current recommendation exists

*minor prescribing recommendations apply due to a paucity of clinical data. Hence the notation of possible effect of PGx on plasma concentration and subsequent dose adjustment consideration. ** There is evidence that CYP2B6 is another major metabolising enzyme for sertraline (14), however the PGx panel utilised for this patient did not at the time of reporting including this variant. *** Unclear clinical significance as both the parent drug and primary metabolite are active. There is insufficient evidence supporting the clinical impact of the decreased O-desmethylvenlafaxine:venlafaxine ratio in CYP2D6 intermediate metabolisers (15). **** Recommendations sourced from CPIC (accessed through PharmGKB) (8). Note exact medication dose adjustments are not readily available. General guidelines are provided for most antidepressants and PGx phenotypes. As with any dose adjustment, careful clinical monitoring for effectiveness and side effects remains imperative. #Note dosages of medications trialled by patient are based on standard prescribing guidelines as recommended in the Australian Therapeutic Guidelines (16).

The patient initially presented with symptoms of depression during hospitalisation, leading to the prescription of the selective serotonin reuptake inhibitor (SSRI) escitalopram at a daily dosage of 10mg and the tetracyclic antidepressant (NASSA) mirtazapine at 15mg nightly, primarily for improving sleep. He experienced nil improvement after a four-week trial. The dose of escitalopram was increased to 20mg without improvement in symptoms following a further six weeks.

He was swapped to sertraline, another SSRI, at a starting dose of 50mg (whilst staying on mirtazapine 15mg at night). After six weeks of treatment, the dose of sertraline was increased to 100mg. The patient did not experience any improvement in his symptoms of depression after another four weeks of treatment at this higher dose.

He was subsequently tapered off sertraline and transitioned to the serotonin and noradrenaline reuptake inhibitor (SNRI) venlafaxine 75mg daily (at the same time he ceased mirtazapine as it was thought that this was not helping and possibly leading to weight gain). After six weeks, the dose of venlafaxine was increased to 150mg daily. The patient noted some alleviation of symptoms after a further 8-week trial of this new dose but not full resolution. He unfortunately experienced side effects of weight gain and sexual dysfunction and so he independently weaned off this medication. At this point the patient was diagnosed as medication-resistant by his treating psychiatrist.

He was subsequently hospitalised for a trial of electroconvulsive therapy (ECT). At this point he had weaned off all anti-depressant medications and was solely taking the benzodiazepine temazepam 10mg at night for insomnia. He undertook 10 rounds of ECT over a course of six weeks. He experienced a partial response; however, this positive response was observed to be short-lived, lasting only a few weeks. At this point he ceased to take temazepam as it was no longer effective in managing his insomnia.

Due to ongoing symptoms of depression, the patient was then started on the SNRI duloxetine 60mg daily. The patient showed some positive response to medication after an eight-week trial but ceased due to reported weight gain and sexual dysfunction.

He was subsequently tapered off and swapped to the serotonin-modulator vortioxetine at a dose of 10mg daily. This was increased to 20mg after 6 weeks due to nil improvement. He did not report any change in symptoms of depression and so ceased this medication due to reported side effect of weight gain.

After a further two months, the patient was hospitalised for 6 weeks for a trial of transmagnetic stimulation (TMS) for a total of 30 sessions. No improvement was observed by the patient or the treating team, leading to the conclusion that it has been unsuccessful for this patient.

Given that the previous trials of antidepressant medications over the course of the last 2 years have yielded limited improvement in his depressive symptoms and in some instances adverse side effects, a PGx panel through blood sampling was undertaken through Sonic Genetics (Australia). At the time of testing only three genes related to antidepressant medications were tested and reported as part of the Sonic Pharmacogenomic Screen: *CYP2D6, CYP2C19*, and *CYP1A2.* Genotyping was performed using Agena MassARRAY genotyping platform. Alleles tested for the three relevant genes are shown in **Table 2** along with the results of the pharmacogenomic analysis for the patient. Note metaboliser status and genotype-phenotype associations were provided by Precision Genetics, USA. CPIC guidelines were used for activity (8, 13).

**Table 2:** Pharmacogenomic analysis.

Gene	Alleles Tested	Genotype	Predicted Phenotype
*CYP2D6*	1, *2, *3, * 4, *5 (gene deletion), *6, *7, *8, *9, *10, *11, *12, *114, *14, *15, *17, *18, *19, *29, * 41, xN (gene duplication)	*1/*4	Intermediate Metaboliser
*CYP1A2*	*1A, *1C, *1F, *1K, *1L, *7, *11	*1A/*1A	Extensive (normal) Metaboliser
*CYP2C19*	*1, *2, *3, *4, *4A, *4B, *5, * 6, *7, *8, *17	*17/*17	Ultrarapid Metaboliser

Alleles listed using the PharmVar Haplotype nomenclature. *Allele. Phenotype predictions are according to those recommend by CPIC which are summarized by respective “diplotype to phenotype translation” tables (available at: https://www.pharmgkb.org/page/pgxGeneRef).

Following PGx testing, it was noted that the patient is an extensive (normal) metaboliser for CYP1A2. The medication agomelatine, a melatonergic agent, is solely metabolised by this enzyme, and it was subsequently thought that this medication would be a good candidate to trial (and could also assist with sleep). It should be noted that there are relatively few reports of PGx relationships for this medication currently.

### 2.1 Patient perspective

After a 6-week trial of this medication at a dosage of 25mg taken at nighttime, the patient reported significantly fewer symptoms of depression as assessed by the GP, had nil residual suicidal ideation, and reported an improvement in sleep. Clinical assessment revealed improvements in insomnia and anxiety. He has not reported to date any side effects. At the time of writing this case study, the patient continues this medication and appears clinically stable. “I want to express my heartfelt gratitude for the care and dedication you’ve shown in helping me find the right antidepressant through pharmacogenomic testing. After years of struggling with multiple medications that either didn’t work or caused terrible side effects, your decision to explore a more personalised approach finally led us to agomelatine. I’m amazed at how much better I feel—my mood is more stable, I’m sleeping better, and I can finally see a light at the end of what felt like a very long tunnel. Thank you for taking the time to truly understand my unique needs, and for giving me hope and relief after so many difficult trials with other treatments.”

The impact of the above CYP polymorphisms on the metabolism of antidepressant medications trialled by the patient and the recommended dose changes is outlined below in **Table 1**.

Furthermore, the pharmacogenomic interaction of all treatment of the patient is depicted below in **Table 3**.

**Table 3:** Pharmacogenomic interaction of all treatment of the patient.

Cytochrome P450			
	CYP2D6 (*)	CYP1A2	CYP2C19 (†)
Escitalopram	S (±), Ih		S (+)
Mirtazapine	S (+)	S (±)	
Venlafaxine (pro-drug)	S (+)		S (±)
Sertraline	S, Ih		S (+)
Duloxetine	S(+), Ih	S (+)	
Vortioxetine	S (+)		S (-)
Agomelatine		S (+)	S

*CYP2D6*1/*4 predicted intermediate metaboliser (IM) phenotype. † CYP2C19*17/*17 predicted ultrarapid metaboliser (UM) phenotype. In (inhibitor). S(Substrate). + (Major Metabolic Pathway), ± (Minor Metabolic Pathway), - (Minor Metabolic Pathway likely not clinically relevant).

## 3 Discussion

This case, involving a mid-20s male with MRD, illustrates the intricate interplay PGx profiling and antidepressant therapy. Although PGx testing provides a pathway toward individualised treatment, the underlying mechanisms remain multifaceted, necessitating both comprehensive genetic interpretation and prudent clinical judgment.

A salient feature of this case is the patient’s classification as an ultrarapid metaboliser for CYP2C19—a genotype with substantial implications for selective serotonin reuptake inhibitors (SSRIs) such as escitalopram and sertraline. In line with earlier work suggesting reduced plasma concentrations and limited therapeutic efficacy in ultrarapid metabolisers (17, 18), this patient did not benefit from standard SSRI doses. Clinical strategies may include dose escalation or selecting alternative agents with less dependence on CYP2C19 (19). However, determining the optimal dose increase requires careful monitoring to prevent side effects associated with a narrow therapeutic window.

Another key consideration is the patient’s intermediate metaboliser status for CYP2D6. CYP2D6 is essential for the metabolism of a number of medications, and intermediate or poor metabolisers have been linked to altered efficacy and a heightened risk of adverse effects (20). The antidepressants venlafaxine and vortioxetine are primarily metabolised by CYP2D6. Venlafaxine provided limited benefit in this case. The ratio of parent drug to its primary metabolite is altered in intermediate metabolisers compared to normal metabolisers, however, the clinical impact of this alteration is unclear (21). B Both venlafaxine and its primary metabolite, desvenlafaxine, are active, but their activities differ. Venlafaxine, a serotonin-norepinephrine reuptake inhibitor (SNRI), has a higher affinity for serotonin reuptake inhibition, whereas desvenlafaxine, also an SNRI, offers a more balanced inhibition of both serotonin and norepinephrine. While both compounds have therapeutic benefits, it is uncertain whether changes in the ratio of venlafaxine to desvenlafaxine affect clinical outcomes. = Some investigators report that venlafaxine’s efficacy can remain satisfactory in intermediate metabolisers if plasma levels are closely monitored (22), even though routine therapeutic drug monitoring is not common in all practice settings. In the case presented, the patient showed little benefit on vortioxetine. Whilst there is limited guidance on genotype-guided dosing for vortioxetine, it has been reported that drug serum concentration varies amongst the different metabolising groups, with poor metabolisers more likely to experience greater serum variation and treatment switch to an alternative antidepressant (23). As noted, this may be a consequence of concentration-dependent adverse effects as the prescribed doses for vortioxetine were similar in all metaboliser groups.

Such variability highlights the importance of a comprehensive PGx evaluation in conjunction with clinical assessments and, where possible, drug-level monitoring.

Further complexity emerged with mirtazapine and duloxetine, both of which are metabolised by multiple metabolic enzymes (CYP2D6, CYP2C19, CYP1A2). This polygenic metabolism can complicate definitive PGx-driven recommendations (24). Hence, broader PGx panels that assess multiple genetic variants may be necessary to draw firm conclusions regarding therapy.

The patient’s positive response to agomelatine, a melatonergic antidepressant primarily metabolised by CYP1A2, is noteworthy. The patient’s normal CYP1A2 metaboliser status likely facilitated efficient clearance, consistent with reports of improved agomelatine efficacy when CYP1A2 metabolism is unimpeded (25). Nevertheless, circadian rhythm regulation and other non-genetic factors may also shape treatment outcomes (26). This underscores the multifactorial nature of depression management, where biological, psychosocial, and environmental influences converge.

Clinically, this case demonstrates how PGx data can help guide antidepressant selection and dosing strategies, particularly in the early management of MRD. However, the limited number of genetic variants assessed in many standard tests underscores the value of broader and more integrated PGx panels that account for the full range of metabolic pathways. This approach aligns with emerging literature advocating the need to expand testing and clinical trials to include data from diverse groups - in order to optimise PGx implementation into clinical practice (24).

In conclusion, this case highlights the benefits of pharmacogenetic profiling—both for clarifying the reasons behind earlier treatment failures and for guiding future therapy. Nonetheless, it also emphasises the need to integrate PGx findings with thorough clinical evaluation, appropriate drug-level monitoring (when feasible), and vigilance for potential adverse effects. As research clarifies genotype–phenotype relationships, clinicians can anticipate a more personalised and effective approach to treating depressive disorders.

This case report is not unique: at least half of patients with depression experience a suboptimal response to their first antidepressant (2), and many go on to trial two or three agents before finding one that provides meaningful symptomatic improvement (2). This suboptimal trajectory often leads to patient disillusionment and an associated increase in suicide risk (27). As such, MRD presents a substantial clinical and economic challenge. Worldwide, MRD is estimated to cost healthcare systems and society billions of dollars in both direct and indirect expenses (28, 29).

The field of PGx holds the potential to revolutionise the management of MRD by offering more precise and personalised medication selection. Nonetheless, immediate clinical utility remains limited for several reasons. First, the repertoire of validated genetic variants implicated in medication response remains small, and ongoing large-scale studies and collaborations are needed to expand the PGx database and refine clinical guidelines. Second, adoption of PGx testing varies widely, affected by differences in cost, infrastructure, and healthcare provider education (24). Rigorous evidence from large-scale clinical trials and real-world data in diverse populations is also critical to strengthen the support for PGx in MRD. Notably, a recent randomised controlled trial employing a 12-gene PGx panel demonstrated promising results for broader implementation (30).

Additionally, longitudinal research is required to explore the long-term benefits and possible limitations of PGx testing in MRD. Such studies will clarify how genetic variants affect relapse, remission rates, and overall durability of response, offering a more comprehensive picture of PGx in the long run.

It should also be recognised that genetic variability is just one of many factors that shape antidepressant outcomes and medication resistance. Environmental factors, substance use, psychological elements, and polygenic interactions may all significantly influence patient responses (4, 12). Incorporating these considerations, together with clinical and demographic data, is essential for a well-rounded approach to patient care.

## 4 Conclusions

Medication-resistant depression (MRD) remains a considerable challenge for both clinicians and patients, demanding an integrative approach that accounts for genetic, biological, and psychosocial factors. Pharmacogenetic (PGx) testing can offer valuable insights into individual responses to antidepressant medications and holds promise for improving MRD treatment outcomes. In this case report, the patient’s genetic variations in CYP2D6, CYP2C19, and CYP1A2 underscore the importance of personalised medicine in enhancing therapeutic efficacy and safety. Tailoring medication selection and dosages based on a patient’s PGx profile can yield significant benefits, yet further research is necessary to cement PGx testing as a standard component of routine clinical practice. Pharmacogenomic (PGx) testing can be performed pre-emptively to guide antidepressant selection, aiming to optimise treatment from the outset rather than in response to therapeutic failure. It should target genes known to affect antidepressant metabolism and response, serving as a complementary tool alongside clinical assessment.

It is also essential to recognise that MRD is a multifactorial condition influenced by variables extending beyond neurotransmission—factors such as early-life trauma and broader psychosocial elements may be underappreciated drivers of treatment resistance. Accordingly, PGx testing should be integrated into a comprehensive treatment framework that addresses these factors, thereby optimising care through a truly personalised approach.

## Data Availability

The original contributions presented in the study are included in the article/**Supplementary Material**. Further inquiries can be directed to the corresponding author.
